# Probing the gut barrier: epithelial permeability as a dynamic marker in eosinophilic gastroenteritis via pCLE

**DOI:** 10.1177/26317745261436496

**Published:** 2026-05-14

**Authors:** Paola Spessotto, Alberto Piacentini, Riccardo Rebuzzi, Diego Rossi, Adrian Zdjelar, Chiara Cigana, Silvia Crestani, Mariagrazia Michieli, Edoardo Vincenzo Savarino, Antonio Di Sabatino, Stefania Maiero, Renato Cannizzaro, Stefano Realdon

**Affiliations:** Molecular Oncology Unit, Centro di Riferimento Oncologico Aviano (CRO), IRCCS, Aviano, Italy; Oncological Gastroenterology Unit, Centro di Riferimento Oncologico Aviano (CRO), IRCCS, Aviano, Italy; Department of Medical, Surgical and Health Sciences, University of Trieste, Trieste, Italy; Oncological Gastroenterology Unit, Centro di Riferimento Oncologico Aviano (CRO), IRCCS, Aviano, Italy; Department of Medical, Surgical and Health Sciences, University of Trieste, Trieste, Italy; Pathology Unit, Centro di Riferimento Oncologico Aviano (CRO), IRCCS, Aviano, Italy; Department of Oncologic Radiation Therapy and Diagnostic Imaging, Centro di Riferimento Oncologico di Aviano (CRO), IRCCS, Aviano, Italy; Hematologic Oncology Unit, Centro di Riferimento Oncologico di Aviano (CRO) IRCCS, Aviano, Italy; Hematologic Oncology Unit, Centro di Riferimento Oncologico di Aviano (CRO) IRCCS, Aviano, Italy; Hematologic Oncology Unit, Centro di Riferimento Oncologico di Aviano (CRO) IRCCS, Aviano, Italy; Division of Gastroenterology, Department of Surgery, Oncology and Gastroenterology, University of Padua, Padua, Italy; Department of Internal Medicine and Medical Therapeutics, University of Pavia, Pavia, Italy; First Department of Internal Medicine, Fondazione IRCCS Policlinico San Matteo, Pavia, Italy; Oncological Gastroenterology Unit, Centro di Riferimento Oncologico Aviano (CRO), IRCCS, Aviano, Italy; Oncological Gastroenterology Unit, Centro di Riferimento Oncologico Aviano (CRO), IRCCS, Aviano, Italy; Department of Medical, Surgical and Health Sciences, University of Trieste, Trieste, Italy; Oncological Gastroenterology Unit, Centro di Riferimento Oncologico Aviano (CRO), IRCCS, Via F. Gallini 2, Aviano 33081, Italy

**Keywords:** barrier dysfunction, eosinophilic gastroenteritis, mucosal integrity, precision endoscopy, probe-based confocal laser endomicroscopy, real-time imaging

## Abstract

Eosinophilic gastroenteritis (EGE) is a rare, chronic inflammatory condition characterized by symptoms and eosinophilic infiltration of the gastrointestinal tract in the absence of secondary causes of gastrointestinal eosinophilia. The advent of probe-based confocal laser endomicroscopy (pCLE) has introduced the possibility of real-time, in vivo microscopic imaging, enabling dynamic assessment of mucosal architecture and barrier function. We report the case of a 32-year-old woman with an atypical manifestation of mucosal EGE, initially presenting with pseudothrombotic microangiopathy secondary to vitamin B12 deficiency. The diagnostic work-up included standard endoscopy, histopathological examination of biopsy samples, and pCLE imaging. pCLE was performed both at baseline and during follow-up to evaluate disease distribution and monitor therapeutic response. Imaging was conducted using the GastroFlex UHD Confocal Miniprobe connected to the Cellvizio system, with images captured within 10 min of intravenous fluorescein administration, digitally stored, and later reviewed by blinded pCLE experts. Initial assessment revealed marked eosinophilic infiltration in the stomach, terminal ileum, and cecum, with corresponding interstitial leakage and inflammatory cell infiltration observed on pCLE. Increased epithelial permeability was also detected in additional intestinal segments lacking histological involvement. After 5 weeks of corticosteroid therapy, the patient achieved clinical and histological remission, accompanied by normalization or significant improvement in pCLE findings. Interestingly, pCLE continued to reveal subtle barrier dysfunction in regions showing histological recovery, highlighting persistent subclinical mucosal alterations. This case underscores the value of pCLE in identifying early intestinal barrier dysfunction and in monitoring therapeutic response in EGE. The concordance between pCLE imaging, histological findings, and clinical outcomes supports its use as a complementary diagnostic and monitoring tool in EGE management.

## Plain Language Summary

Seeing the unseen: detecting hidden inflammation in a rare gastrointestinal disorder

Eosinophilic gastroenteritis (EGE) is a rare condition where certain white blood cells build up in the digestive tract, causing inflammation and symptoms like abdominal pain, vomiting or diarrhea. We report the case of a 32-year-old woman with an unusual form of EGE, first presenting with signs of vitamin B12 deficiency.

To help diagnose and monitor the disease, we used a special imaging technique called probe-based confocal laser endomicroscopy (pCLE), which allows real-time microscopic views of the gut lining. This tool revealed signs of inflammation and increased gut permeability, even in areas that looked normal under the microscope.

After treatment, the patient improved, but pCLE still showed subtle changes, suggesting ongoing low-level damage. This case shows how pCLE can help detect hidden inflammation and monitor treatment response in EGE.

## Introduction

Eosinophilic gastroenteritis (EGE) is a rare and chronic inflammatory disorder characterized by symptoms and pathological eosinophilic infiltration of the gastrointestinal (GI) tract in the absence of identifiable secondary causes of gastrointestinal eosinophilia. It is classified into mucosal, muscular, and serosal subtypes based on the depth of tissue involvement.^1^

In recent years, epidemiological data have demonstrated a rising incidence and prevalence of eosinophilic gastrointestinal diseases (EGIDs), including EGE. A large population-based study from Israel showed that the incidence of EGIDs has tripled over the past decade, increasing from 2.51 to 7.88 per 100,000 person-years, with a similar trend noted for nonesophageal EGIDs such as EGE.^2^ Similarly, data from the United States have confirmed increasing diagnostic rates over a 10-year period across multiple centers,^3^ with prevalence estimates for EGE reaching 8.4 per 100,000 individuals.^1^

Although the exact pathophysiologic mechanism of noneosinophilic esophagitis EGID beyond eosinophilic involvement remains unclear, current research highlights the multifactorial roles of IgE—mediated and T-helper 2—mediated responses.^4^

Diagnosis is based on the presence of GI symptoms, histological confirmation of eosinophilic infiltration in one or more segments of the GI tract, and exclusion of other causes of tissue eosinophilia.^5^

Standard treatment typically involves systemic corticosteroids, while dietary modifications and immunomodulators are often employed for long-term management.^6^ Long-term strategies seek to maintain remission while minimizing the adverse effects associated with prolonged corticosteroid use. The therapeutic goals in EGE remain poorly defined. In clinical practice, symptom control remains the primary objective. GI symptoms such as abdominal pain, diarrhea, nausea, and vomiting significantly impair patients’ quality of life, and their resolution is considered the most meaningful therapeutic endpoint.^7^ Beyond symptom control, histological remission is often considered a secondary goal.^4^

Given the rarity of EGE and the limited literature, the case reported in this study represented a valuable opportunity to combine conventional diagnostics with probe-based confocal laser endomicroscopy (pCLE). This minimally invasive technique allows real-time, in vivo microscopic evaluation of the GI wall architecture, enabling the exploration of potential correlations among endomicroscopic, endoscopic, and histologic findings.^8^ Notably, pCLE has recently been validated as a tool for dynamic structural and functional assessment of mucosal barrier integrity by visualizing crypt architecture, vascular tortuosity, and fluorescein leakage. pCLE has been effectively utilized in the context of IBD, where a hallmark feature is the disruption of intestinal epithelial barrier integrity, leading to altered permeability, impaired homeostasis, and a dysregulated immuno-inflammatory environment.^9^ When combined with intravenous fluorescent agents, pCLE enables up to 1000-fold magnification of the mucosa, allowing detailed visualization of crypt architecture, vessel tortuosity, and fluorescein leakage. This high-resolution imaging can support the prediction of therapeutic response and potential adverse clinical outcomes. Therefore, assessing the intestinal barrier is a valuable tool for detecting GI tissue damage and dysfunction.

## Case description

A 32-year-old woman was admitted to the Onco-Hematology Unit of our Comprehensive Cancer Center (CRO IRCCS, Aviano) with suspected leukemia, prompted by the detection of trilinear cytopenia (Hb 6 g/dL, MCV 122 fL, platelets 120 × 10^3^/mm^3^, WBCs 2510/mm^3^) during evaluation for chronic asthenia that had progressively worsened over several months. Further hematologic workup revealed a diagnosis of pseudothrombotic microangiopathy secondary to severe vitamin B12 deficiency. The patient also reported acute diarrhea, dyspepsia, recent unintentional weight loss, and a history of recurrent anemia (Supplemental Material).

To investigate the etiology of the vitamin B12 deficiency and assess her GI symptoms, a comprehensive gastroenterological evaluation was performed. Laboratory tests showed absence of antiparietal cell antibodies (APCA), elevated gastrin-17 levels (17 pmol/L), reduced pepsinogen I (5.8 µg/L), and normal pepsinogen II levels, yielding a pepsinogen I/pepsinogen II ratio of 1.23—finding consistent with corpus-predominant atrophic gastritis. Stool studies were negative for bacterial, fungal, and parasitic infections. Serological testing for celiac disease was negative. Thyroid function tests revealed mildly elevated TSH (5.64 µIU/mL) with normal free T4 levels (fT4: 0.95 ng/dL). Fecal calprotectin was slightly elevated (68 µg/g).

**Figure 1.**
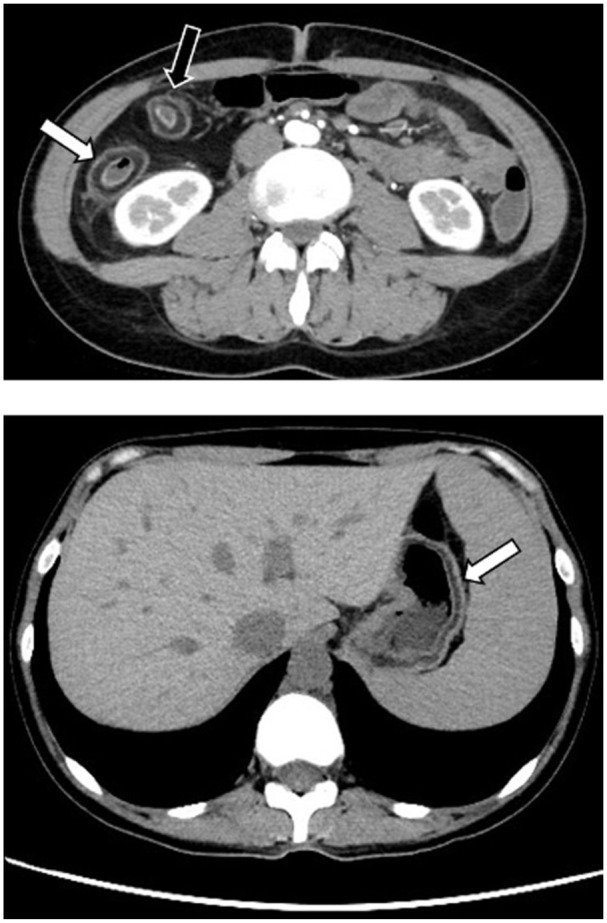
Axial CT section before treatment. The upper section of CT scan shows moderate bowel wall thickening with submucosal fat infiltration involving the ascending (white arrow) and proximal transverse colon (black arrow). In the lower section, there is also thickening of the gastric greater curvature.

A thoracoabdominal CT scan showed moderate bowel wall thickening with submucosal fat infiltration involving the ascending and proximal transverse colon (Figure 1, black and white arrows, respectively, in the upper image). Similar fatty infiltration was observed in the submucosal of the stomach’s greater curvature (Figure 1, white arrow in the lower image).

To further assess the suspected atrophic gastritis associated with B12 deficiency and to investigate the patient’s diarrhea and radiologic findings, both esophagogastro-duodenoscopy (EGDS) and colonoscopy were performed, including pCLE.

Based on initial endoscopic and histological findings, a diagnosis of mucosal EGE affecting the stomach, terminal ileum, and cecum was established. Additional laboratory tests revealed normal total IgE levels and negative allergen-specific IgE on RAST analysis. Following diagnostic confirmation, the patient began a 9-week course of corticosteroid therapy with prednisone.

Approximately 12 weeks after diagnosis (5 weeks after starting prednisone), a comprehensive follow-up, including repeat endoscopy with pCLE, was conducted to objectively assess treatment response.

## Methods

Real-time pCLE images was performed using the GastroFlex UHD Confocal Miniprobe connected to the Cellvizio system (Cellvizio, Mauna Kea Technology, Paris, France), which operates at a 488 nm excitation wavelength. The procedure was conducted during gastroscopy and colonoscopy (Olympus series 180). The Miniprobe was introduced through the instrument’s working channel, with its distal tip positioned perpendicularly to the target mucosal surfaces. Images were acquired within the first 10 min following intravenous administration of fluorescein (3 mL of a 10% saline solution). To ensure a representative sampling of the dynamic mucosal interface, each segment was examined for a minimum of 2 min. During this time, at least three separate, stable video sequences (15–30 s each) were captured after achieving gentle tissue contact and clear visualization. Sequences with significant motion artifact were discarded and reacquired.

For offline analysis, a minimum of 50 interpretable frames per segment were extracted from the stored video sequences. These frames were reviewed using the Cellvizio Viewer software (Mauna Kea Technologies, Paris, France) and presented in a randomized order to two blinded expert reviewers for independent assessment. The interpretation model was designed as a consensus process to generate a single, best-estimate classification. For defining permeability, we used a binary (present/absent) assessment based on the visual identification of fluorescein efflux into the lumen. For patterns like “bright lumen,” we relied on qualitative descriptors common in key pCLE literature.^10^ All videos with discrepant reads were then reviewed in a joint session with discussion until a consensus diagnosis was reached. The raw percent agreement between the two pCLE readers prior to consensus was 90%. The final consensus read was used for all correlation analyses with clinical/histologic data.

Relevant Equator (in particular the CAREreporting)^11^ guidelines have been followed in this study.

## Diagnostic assessment

### Baseline EGDS

**Figure 2.**
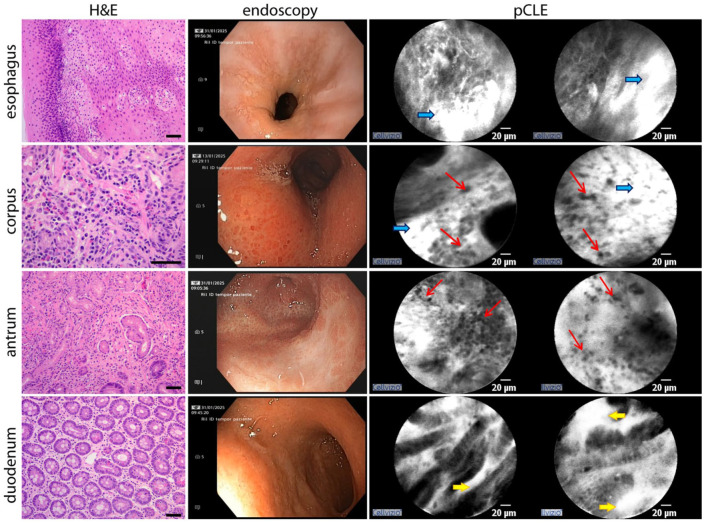
Baseline EGDS macroscopical, pCLE and hystological findings compared; in pCLE images, red arrows indicate eosinophilic infiltration, yellow arrows show epithelial permeability and blue arrows highlight vascular permeability. Eosinophilic infiltration is most prominent in both pCLE and histology. Scale bar in H&E images, 50 μm. EGIDs, eosinophilic gastrointestinal diseases; pCLE, probe-based confocal laser endomicroscopy.

Macroscopic examination of the esophagus revealed no pathological abnormalities. pCLE imaging showed minimal vascular leakage (Figure 2, blue arrows), and histological analysis confirmed the absence of any inflammatory infiltrates.

The gastric mucosa displayed diffuse atrophy with numerous small, raised, hyperemic areas throughout the stomach, sparing only a dyschromic region located along the greater curvature between the antrum and body. pCLE revealed increased permeability and interstitial cellular infiltration across the gastric mucosa (Figure 2, thin red arrows). Histological examination confirmed mucosal atrophy with marked eosinophilic infiltration (>30 eosinophils/HPF in 5 HPFs). In the dyschromic area between the antrum and body, pCLE identified a squamous-like pattern; corresponding histology revealed squamous metaplasia.

The duodenal mucosa appeared macroscopically normal. However, pCLE detected mildly altered glandular mucosa with increased interstitial leakage (Figure 2, yellow arrows). Histological analysis showed mild chronic inflammation with preserved villous architecture and no significant eosinophilic infiltration (Figure 2).

### Baseline colonoscopy

**Figure 3.**
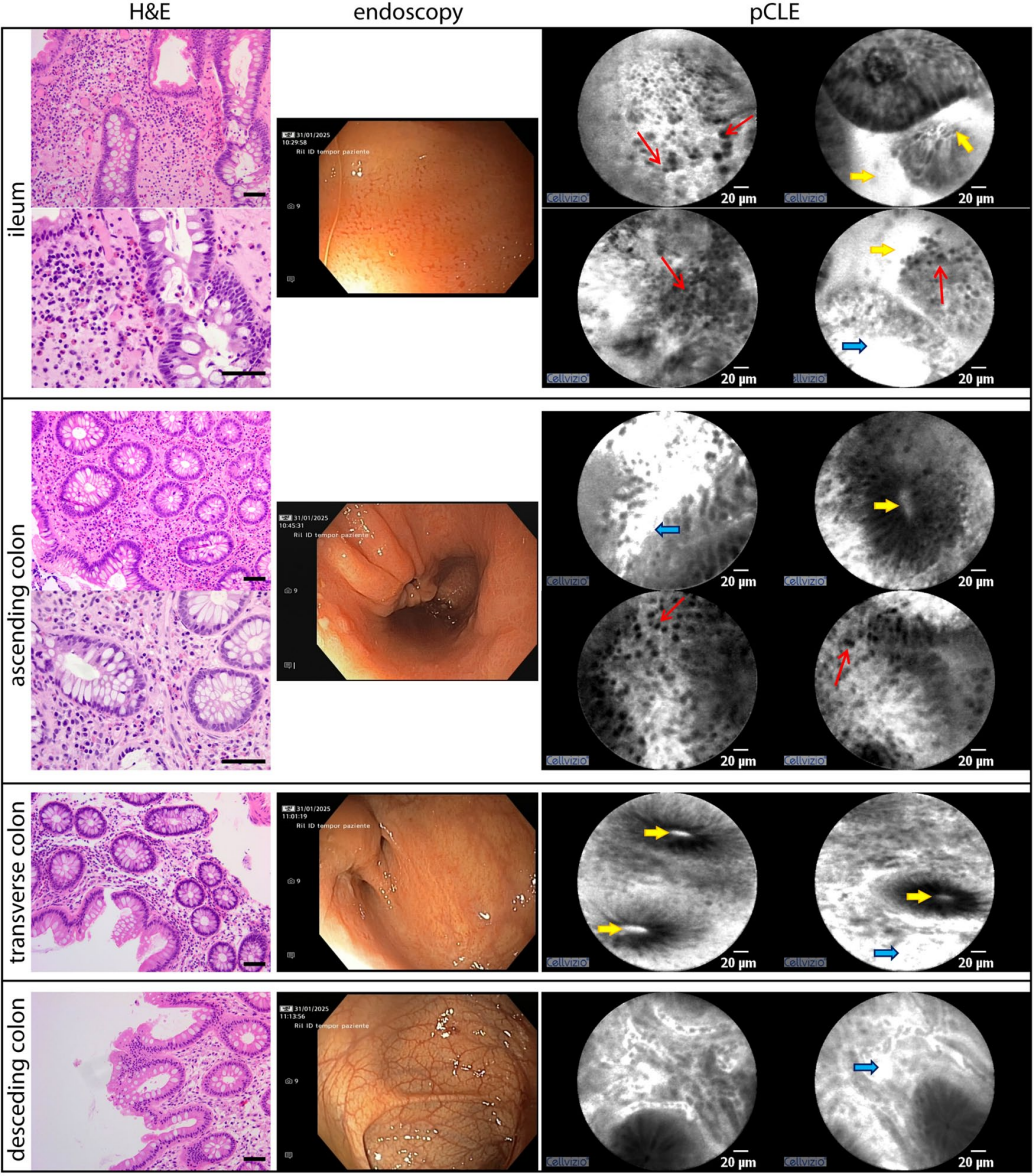
Baseline colonoscopy macroscopical, pCLE and hystological findings are compared. Eosinophilic infiltration (red arrows), epithelial permeability (yellow arrows) and vascular permeability (blue arrows) are predominantly observed in the ileum and ascending colon on pCLE, correlating with the presence of eosinophilic infiltration confirmed by histology. Scale bar in H&E images, 50 μm. pCLE, probe-based confocal laser endomicroscopy.

The terminal ileum mucosa appeared hyperemic, with multiple superficial erosions, predominantly affecting the distal segment. pCLE imaging revealed disrupted glandular architecture, increased interstitial leakage, and marked interstitial cellular infiltration. Histology confirmed mild chronic inflammation with preserved villous architecture, superficial erosions, and significant eosinophilic infiltration (>56 eosinophils/HPF; Figure 3).

The right colon mucosa was hyperemic and edematous, with attenuated vascular pattern. pCLE showed irregular glandular structures and intraluminal glandular accumulation of contrast (Figure 3, bright lumen pattern, yellow arrows), indicating increased epithelial permeability. Interstitial cellular infiltration was most pronounced in the cecum. Histological analysis of cecal samples showed crypt hyperplasia and marked eosinophilic infiltration (>100 eosinophils/HPF). In contrast, ascending colon biopsies showed preserved crypt architecture and subthreshold eosinophilic infiltration (<100 eosinophils/HPF).

The transverse colonic displayed mild inflammatory changes that diminished progressively in the distal direction. Near the hepatic flexure, a substenotic segment separated inflamed bowel from macroscopically normal tissue. pCLE imaging revealed generally preserved glandular architecture, with occasional bright lumens. Histology confirmed a gradual decrease in inflammatory activity distally, with chronic inflammation of the lamina propria and no significant eosinophilic infiltration (<84 eosinophils/HPF).

The left colon appeared endoscopically normal. pCLE revealed no pathological alterations, and the bright lumen pattern was absent. Histological examination showed only mild, nonspecific chronic inflammation of the lamina propria.

### Ongoing treatment EGDS

Endoscopic examination of the esophagus revealed no inflammatory changes with neither pCLE nor histological analysis detecting any abnormalities.

**Figure 4.**
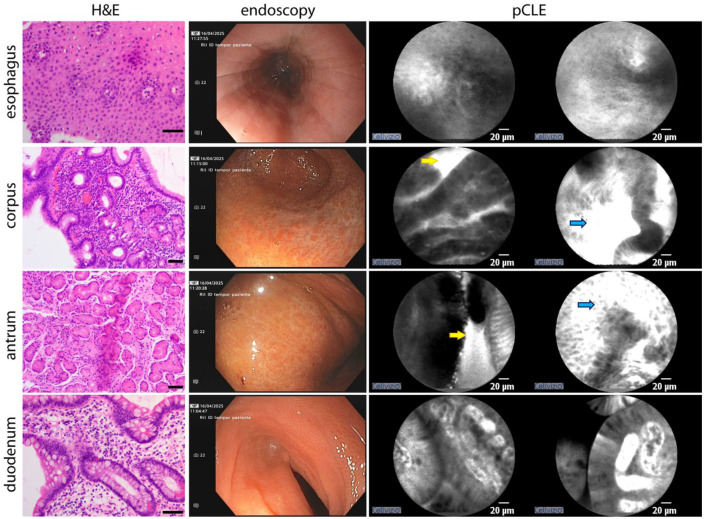
Macroscopic, pCLE, and hystological findings during ongoing treatment (EGDS). Although some disease features remain macroscopically visible, only mild or sporadic pathological alterations are detected by pCLE, with limited histological abnormalities. Scale bar in H&E images, 50 μm. EGIDs, eosinophilic gastrointestinal diseases; pCLE, probe-based confocal laser endomicroscopy.

The gastric mucosa showed features of atrophy; however, notable mucosal healing was evident, including progressive resolution of previously described hyperemic areolas. pCLE revealed only mild interstitial leakage, with no evidence of cellular infiltration. Histology confirmed the absence of eosinophilic infiltrates. Both the first and second portions of the duodenum appeared macroscopically normal and histologically intact, with pCLE imaging showing no evidence of interstitial leakage (Figure 4).

### Ongoing treatment colonoscopy

The terminal ileum appeared macroscopically normal. pCLE imaging displayed only mildly reduced interstitial leakage, with no evidence of interstitial cellular infiltration. Histological analysis confirmed the absence of eosinophilic infiltrates.

The mucosa of the cecum and ascending colon appeared intact, with a marked reduction in mucosal inflammation. No ulcerations were observed, and partial restoration of the vascular pattern was noted. pCLE revealed minimal interstitial leakage at the cecum and “dark lumens” in the ascending colon, without signs of cellular infiltration. Histological examination confirmed the absence of significant eosinophilic infiltration.

**Figure 5.**
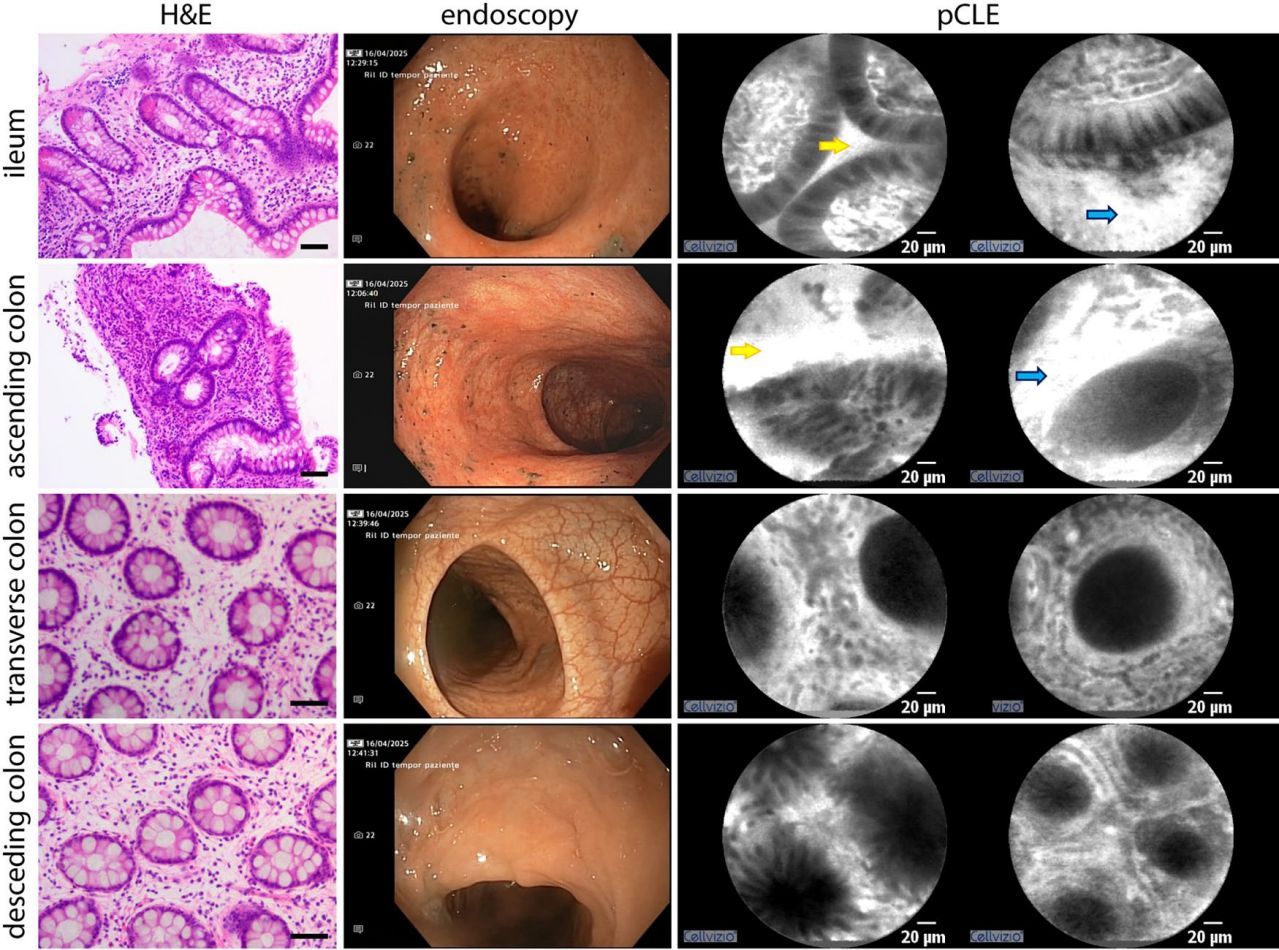
Macroscopic, pCLE and hystological findings during ongoing treatment (colonoscopy). A significant degree of mucosal healing is evident at macroscopic evaluation, accompanied by a marked reduction in pCLE-detected pathological features and the complete resolution of eosinophilic infiltration on histological analysis. Scale bar in H&E images, 50 μm. pCLE, probe-based confocal laser endomicroscopy.

The transverse and left colon showed normal mucosa on both macroscopic and histological evaluation. No “bright lumens” or interstitial cellular infiltration were detected by pCLE in any of the segments examined. These findings are illustrated in Figure 5, which summarizes the macroscopic, pCLE, and histological features observed across the examined intestinal segments.

The patient did not report any discomfort or experience complications during the endoscopic procedures, despite their extended duration compared to routine exams. Following the initiation of corticosteroid therapy, she experienced a notable improvement in both GI symptoms and overall well-being.

## Discussion

### Atypical presentation of mucosal EGE

Correlation of endoscopic and histological findings with the patient’s clinical history supported a diagnosis of mucosal EGE. However, several atypical features were noted. The patient did not report abdominal pain—typically considered a hallmark symptom of mucosal EGE-and had no personal or family history of atopy. Notably, she developed pseudothrombotic microangiopathy secondary to severe vitamin B12 deficiency, an uncommon presentation, as EGE is more frequently associated with microcytic iron-deficiency anemia.^12^

### Intestinal barrier dysfunction as an early marker of disease activity in EGE

Biopsy specimens from the stomach, terminal ileum, and cecum revealed significant eosinophilic infiltration, correlating with pCLE findings of both interstitial leakage and cellular infiltration, as well as with macroscopic evidence of inflammation in these regions. Interestingly, pCLE also identified increased epithelial permeability in the esophagus, duodenum, ascending colon, and transverse colon, even in the absence of histological eosinophilic infiltration. Among these, only the ascending and proximal transverse colon exhibited endoscopic signs of inflammation; the esophagus, distal transverse colon, and left colon appeared macroscopically normal.

These findings suggest that pCLE may detect intestinal barrier dysfunction in EGE before histologic and, in some cases, even endoscopic abnormalities become evident. This supports the potential role of pCLE as a sensitive tool for identifying subclinical disease activity and highlights its value in the early assessment and monitoring of EGE.

### Restoration of barrier function as a therapeutic target in EGE

Following 5 weeks of prednisone therapy, cellular infiltration had resolved in regions where both epithelial permeability and eosinophilic infiltration were previously detected (i.e., stomach, terminal ileum, and cecum), although mild residual permeability was still observed. In contrast, segments that had initially shown only increased permeability (duodenum, ascending colon, transverse colon, and esophagus) exhibited complete normalization on pCLE, with no evidence of interstitial leakage. These findings highlight the responsiveness of epithelial barrier dysfunction to corticosteroid therapy. Notably, in areas where histological eosinophilia had resolved and mucosal healing was evident, interstitial leakage on pCLE often persisted as the sole remaining abnormality. The observed concordance between clinical improvement, histological resolution, and normalization of pCLE findings underscores the potential role of intestinal barrier restoration as both a marker of therapeutic response and a therapeutic target itself. As such, pCLE may serve not only as a diagnostic and monitoring tool for but also a guide in optimizing treatment strategies for EGE.

### Limitations of the case report

As previously reported, our patient’s clinical presentation, medical history, and endoscopic findings were notably atypical for mucosal EGE, potentially limiting the generalizability of our observations.

Furthermore, the interpretation of pCLE images currently relies heavily on the experience and subjective judgment of the endoscopist, leading to variability in reporting and limiting reproducibility across different centers. The absence of standardized criteria for image evaluation further restricts the broader clinical application of this technique. Recognizing this as a key limitation, we emphasize that future research must prioritize the development and rigorous validation of robust quantitative or semi-quantitative scoring systems. The establishment of such standardized metrics is essential to enhance diagnostic objectivity, improve interobserver reproducibility, and facilitate reliable longitudinal monitoring in clinical practice.

Our study design was primarily focused on the correlation between in vivo pCLE patterns and traditional histologic inflammation (eosinophils). Protein-level analysis of ZO-1, occludin, or specific claudins would be ideal to strengthen the barrier function hypothesis with mucosal tight junction markers. Future studies incorporating molecular analyses of epithelial junction proteins are warranted to provide direct biological validation of the barrier defects suggested by pCLE.

Finally, the limited number of patients studied with pCLE in the context of EGE, combined with the low prevalence of the disease and the restricted availability of pCLE equipment in most clinical centers, poses significant challenges to widespread adoption. Therefore, we advocate for broader implementation of pCLE in patients diagnosed with or suspected of having EGE, to support future efforts in validation, standardization, and clinical integration of this promising technique.

## Conclusion

While pCLE has been widely recognized for its applications in oncology—particularly in the assessment of angiogenesis—^13–15^ it is increasingly demonstrating value in the evaluation of nonneoplastic GI diseases. This imaging technique offers real-time, high-resolution visualization of the mucosa and vasculature, enabling the assessment of key features such as crypt architecture, vessel morphology, and vascular permeability. As such, pCLE has emerged as a powerful tool for studying intestinal barrier integrity across a range of GI disorders.

Beyond its established role in cancer, recent studies have highlighted the utility of pCLE in inflammatory conditions, including IBD disease and, more recently, eosinophilic GI disorders. In our very recent publication on EoE, we demonstrated that pCLE, using vascular permeability as a functional marker, enabled dynamic monitoring of mucosal response to therapy.^16^ The observed correlation between pCLE findings, disease activity, and treatment response underscores its potential as a complementary tool in the clinical management of EGE. Notably, the persistence or resolution of epithelial leakage may serve as a promising biomarker for mucosal healing and therapeutic efficacy in eosinophilic and other inflammatory GI diseases.

The functional parameters assessed by pCLE show promise for evaluating therapy response in clinical research; however, its role in routine clinical monitoring remains limited by practical constraints including cost, availability, and operator dependence. As such, its role is not for broad screening but as a targeted diagnostic tool for complex cases. Cost-effectiveness depends on its ability to provide definitive answers where conventional methods fail, preventing unnecessary follow-up tests and treatments. Future work will be contingent on demonstrating that the unique diagnostic information provided by pCLE improves patient outcomes sufficiently to justify the added resource utilization.

## Supplemental Material

sj-pdf-1-cmg-10.1177_26317745261436496 – Supplemental material for Probing the gut barrier: epithelial permeability as a dynamic marker in eosinophilic gastroenteritis via pCLE Supplemental material, sj-pdf-1-cmg-10.1177_26317745261436496 for Probing the gut barrier: epithelial permeability as a dynamic marker in eosinophilic gastroenteritis via pCLE by Paola Spessotto, Alberto Piacentini, Riccardo Rebuzzi, Diego Rossi, Adrian Zdjelar, Chiara Cigana, Silvia Crestani, Mariagrazia Michieli, Edoardo Vincenzo Savarino, Antonio Di Sabatino, Stefania Maiero, Renato Cannizzaro and Stefano Realdon in Therapeutic Advances in Gastrointestinal Endoscopy

## Declarations

**Ethics approval and consent to participate:** Ethical approval to report this case was obtained by the Ethics Committee of the CRO-IRCCS of Aviano (Institutional review board number: CRO-2019-62), and the patient provided written informed consent.

**Consent for publication:** We have obtained written informed consent to publish from the patient.

**Availability of data and materials:** Not applicable.
